# Comprehensive Transcriptomic and Proteomic Analysis of Severe Pressure Ulcer Patients Identifies Molecular Signatures Associated with Impaired T Cell Function

**DOI:** 10.3390/biom15121682

**Published:** 2025-12-02

**Authors:** Kanhaiya Singh

**Affiliations:** Department of Surgery, McGowan Institute for Regenerative Medicine, University of Pittsburgh, Pittsburgh, PA 15219, USA; singhk@pitt.edu; Tel.: +1-412-624-2449

**Keywords:** pressure ulcer, diabetes, transcriptomics, proteomics, T cell, inflammation

## Abstract

Pressure ulcers (PUs) result from prolonged pressure and shear forces, which cause local skin and soft tissue injury. Elderly patients with pressure injuries face a higher risk of death. Diabetes presents a significant comorbid condition that increases the risk of PU development due to underlying neuropathy, vasculopathy, and impaired wound healing. Recent molecular biology research on PU subjects has identified inappropriate responses to inflammatory stressors as a significant risk factor. Systemic manifestations, such as an increased abundance of inflammatory cells and alterations in inflammatory mediators, have been linked to PU formation. The present study adopted a bioinformatics, multi-omic data-mining approach to understand cellular and molecular dysregulation and identify biomarkers that may guide the development of more effective screening, diagnostic, and therapeutic strategies in the management of severe PU subjects. At the RNA level, differential gene expression indicated T cell dysfunction and impaired T cell communication in severe PU subjects. Protein-based analysis further validated this finding, as T lymphocyte functional readouts, such as Th1 cell response, memory T cell activation, and Th17 cell differentiation, were predicted to be downregulated. Taken together, our results show that T lymphocyte function and communication remain impaired in severe PU and could guide the development of a therapeutic cell-based treatment for regenerative medicine.

## 1. Introduction

Pressure ulcers (PUs), also known as bedsores or decubitus ulcers, result from prolonged pressure and shear forces, leading to local skin and soft tissue injury [1,2]. PUs extend over bony prominences such as the sacrum, ischium, heel, and greater trochanter [3]. Clinically, PUs affect thousands of people each year, resulting in an estimated economic burden of more than USD 26.8 billion [4,5]. The global prevalence of PUs reported in hospitalized patients is 12.8%, with an incidence rate of 5.4 per 10,000 patient days [6]. Based on the severity of the ulcers, PUs can be classified into grades I–IV [7,8]. Grades I–II PUs include developing ulcers that can be addressed and reversed if the underlying causes are adequately managed. Grades III–IV PUs, on the other hand, are severe and involve substantial injury to the subcutaneous tissues, including muscle or bone [9]. Elderly patients with pressure injuries have a higher risk of death [hazard ratio (HR) = 1.78, 95% confidence interval (CI) = 1.46 to 2.16] [10]. The risk increases further when the patient is suffering from severe PUs (grade III–IV; HR: 2.41; 95% CI: 1.08–5.37), a condition that may serve as a predictor of mortality in the elderly owing to underlying poor health, immobility, and frailty.

A combination of extrinsic (such as pressure, friction, shear force, moisture, etc.) and intrinsic (endothelial dysfunction, inflammation, ischemia, malnutrition, anemia, etc.) factors has been shown to make an individual susceptible to developing PUs [11]. Diabetes presents a significant comorbid condition that increases the risk of PU development due to underlying neuropathy, vasculopathy, and impairment in wound healing [12,13,14,15,16,17,18,19]. In 2017, a meta-analysis of 24,114 individuals across 16 studies indicated that diabetic patients undergoing surgery have a higher risk of developing PUs [odds ratio (OR) = 1.77, confidence interval (CI) = 1.45 to 2.16] [19]. Similar findings were observed in another study conducted in 19,724 patients, which demonstrated that diabetes increased the risk of surgery-related pressure ulcers by 1.5 times compared with non-diabetic patients (CI = 1.25 to 1.85) [20].

Recent molecular biology research on PU subjects has revealed that an inappropriate response to inflammatory stressors is a significant risk factor [21]. Notably, (i) prolonged unresolved inflammation, (ii) an imbalance in the ratio of matrix metalloproteinases and tissue inhibitors of metalloproteinases, (iii) stem cell dysfunction, and (iv) reduced presence and activity of growth factors have been attributed to diabetic and non-diabetic PUs [22]. Systemic manifestations, such as an increase in the abundance of inflammatory cells, as well as changes in inflammatory mediators, have also been shown to be associated with PU formation [23]. Among the cellular deregulations, the most reported include excessive presence of neutrophils, the presence of senescent fibroblasts, T cell anergy, or tolerance [24,25]. To clarify the complex role exerted by all these mediators in this process, the combination of molecular approaches such as transcriptomics and proteomics is now being utilized to obtain a holistic understanding of cellular mechanisms in PU subjects [26,27]. The present study adopts a bioinformatics, multi-omic data-mining approach to understand the cellular and molecular dysregulation in PU subjects. Identifying systemic or tissue-level molecular biomarkers can help improve screening, diagnostic, and treatment strategies for managing severe PU patients [27,28].

## 2. Materials and Methods

### 2.1. RNA Sequencing Data Download and Analysis

Serum RNA sequencing data available in the GEO database under accession number #GSE230161 were downloaded [26]. The dataset contained RNA sequencing data of peripheral blood mononuclear cells (PBMCs) obtained from a total of 20 patients (*n*  =  10 patients with grade II PUs and *n*  =  10 patients with grade III–IV PUs) [26] (Table S1). RNA-seq was performed on DNase I-treated RNA samples with a RIN > 8 (RNA integrity number) [26]. RNA-seq libraries were generated using the Next^®^ Ultra II Directional RNA Library Prep Kit (New England BioLabs, Ipswich, MA). Sequencing was performed on the Illumina HiSeq 2500 instrument to generate a dataset (minimum of 8 M reads per sample) at 50-nucleotide read length in single-end format (1  ×  50) [26].

FASTQ files obtained after sequencing were preprocessed by removing rRNA sequences [26] using SortMeRNA 2.1. Next, adapters were trimmed and low-quality sequences were removed [26] using BBMap version 38.92 and Cutadapt 1.15. Next, reads were aligned to GRCh38.p13 (NCBI) using STAR 2.7.10b software.

As an additional step, the entire dataset was re-normalized using the average expression of alpha-1-B glycoprotein (*A1BG*) in controls. Next, log2 fold change was calculated for genes significantly different (*p*-value < 0.05, Student’s *t*-test) between grade III–IV vs. grade II PU. The differentially expressed genes (DEGs) were subjected to downstream pathway analysis using the Ingenuity Pathway Analysis (IPA) tool. To understand the impact of diabetes, a within-group comparison of DEGs was performed in severe (grade III–IV) PU subjects (diabetic versus non-diabetic).

### 2.2. Proteomics Data Download and Analysis

Differentially expressed proteins in severe PU (grade III–IV) tissue (*n* = 6) vs. control tissue (*n* = 6), as documented by Baldan-Martin et al., 2020, were analyzed [27]. The study design, sample collection, and proteomics analysis as provided by the authors [27] are presented below.

Two different samples were collected from each patient: (i) PU tissue and (ii) adjacent tissue used as a control. Quantitative differential LC-MS/MS analysis was performed using tandem mass spectrometry [27,29,30]. Briefly, TMT 10-plex isobaric labeling was performed, and the labeled peptides were analyzed by LC-MS/MS using a C-18 reversed-phase nano-column [27]. Peptide identification was performed using the probability ratio method, and the false discovery rate (FDR) was calculated [27]. Statistical analysis of the quantitative data was carried out using the weighted spectrum peptide and the protein (WSPP) statistical model [27].

For statistical analysis, the authors considered proteins differentially expressed if: (i) they were identified with at least two peptides and (ii) they had log2 ratios expressed in the form of the standardized variables (Zq) ±1.5 (*p*  ≤  0.05) [27]. The changes in peptide and protein abundance were assessed with a 1% FDR, using the TMT reporter ion intensities from MS/MS scans from SanXoT software as inputs to the WSPP model [27,31].

### 2.3. Ingenuity Pathway Analysis

The significant transcripts and proteins obtained were analyzed using the Ingenuity Pathway Analysis (IPA) tool [32,33,34]. Core analysis was then performed to identify enriched pathways represented by differentially expressed genes or proteins using the Ingenuity Knowledge Base. The IPA-derived z-score predicted the direction of change for the function (+z = activation, −z = inhibition). Graphical representations of the biological relationships between differentially expressed genes or proteins are presented.

## 3. Results

**Figure 1:**
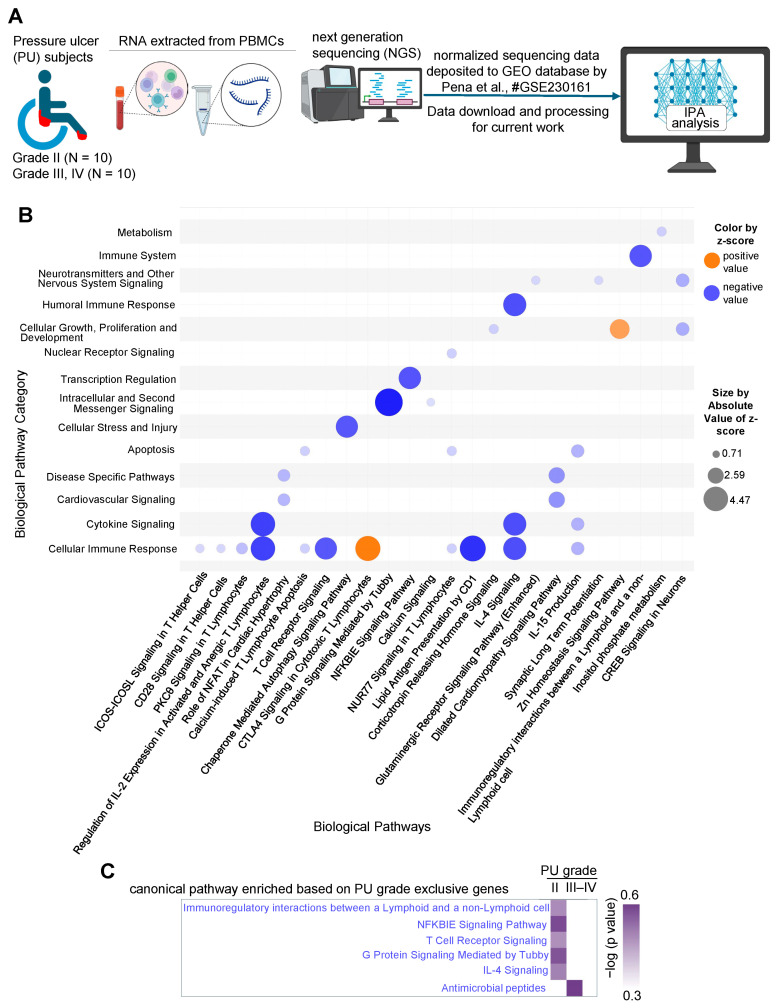
(**A**) Schematic diagram showing the layout of the transcriptomics study conducted from the dataset deposited in the GEO database under accession number GSE230161 [26]. Created in BioRender. Ingenuity Pathway Analysis (IPA) was performed to investigate enriched pathways and upstream regulators based on the differentially expressed genes. (**B**) Bubble chart created with IPA software representing the significant biological pathways (*x*-axis) and their broad categories (*y*-axis) enriched by the differentially expressed genes in severe PU (grades III–IV) as compared to grade II PU. The intensity of the color represents the magnitude of pathway being up- or down-regulated. (**C**) Comparison analysis of T cell communication-related pathways enriched in A in differentially expressed genes exclusively present in either severe PU or grade II PU using the comparison analysis function of IPA.

The transcript profiles of PBMCs isolated from patients with severe PUs (grades III–IV) versus grade II PU were analyzed to catalog the immune modulation of inflammatory responses. Normalized RNA sequencing data from grade II (N = 10) and grades III–IV (N = 10) were retrieved from GEO (accession ID = GSE230161) (Figure 1A, Table S1). A total of 791 differentially expressed (DE) transcripts (*p* < 0.05) that were significantly different between grade II PU and severe PU subjects were then examined for differences in their fold changes. Out of these 791 DE transcripts, 65 were exclusively expressed in only one condition (20 in severe PU, 35 in grade II PU). The remaining 726 DE transcripts were analyzed using IPA to investigate: (i) canonical pathways, (ii) gene-networks, (iii) disease and biofunction enrichment, and (iv) upstream regulators associated with impaired modulation of inflammatory responses in severe PU subjects.

Out of the 726 DE transcripts obtained earlier, 683 analysis-ready differentially expressed genes (DEGs) were identified in IPA (399 downregulated and 284 upregulated) and were used for downstream analysis. First, significant biological pathways and their broad categories were analyzed (−log(*p*-value) > 2 and absolute z-score > ±0.5, Table 1) and visualized as a bubble chart (Figure 1B). The cellular immune response pathway category was found to be downregulated in severe PU subjects (Figure 1B). The significant downregulated pathways (based on activation z-score) in this category included: (i) regulation of IL-2 expression in activated and anergic T lymphocytes, (ii) calcium-induced T lymphocyte apoptosis, (iii) lipid antigen presentation by CD1, (iv) chaperone-mediated autophagy, and (v) IL-15 production, demonstrating T cell dysfunction (Figure 1B, Table 2). Additionally, compromised T cell communication was also observed in severe PU subjects, as marked by significant downregulation of pathways including (i) T cell receptor signaling, (ii) IL-4 signaling, (iii) immunoregulatory interactions between a lymphoid and non-lymphoid cell, (iv) NFKBIE signaling, and (v) G protein signaling mediated by Tubby (Figure 1B, Table 2). This finding was further strengthened when genes expressed exclusively in one condition (20 in severe PU, 35 in grade II PU) were analyzed. Genes related to T cell communication pathways were enriched in grade II PU, while severe-grade PU was enriched only by antimicrobial peptide-related genes (Figure 1C). Among the pathways that were upregulated in severe PU subjects were (i) CTLA4 signaling in cytotoxic T lymphocytes and (ii) Zn homeostasis signaling pathway (Figure 1B, Table 2).

**Figure 2:**
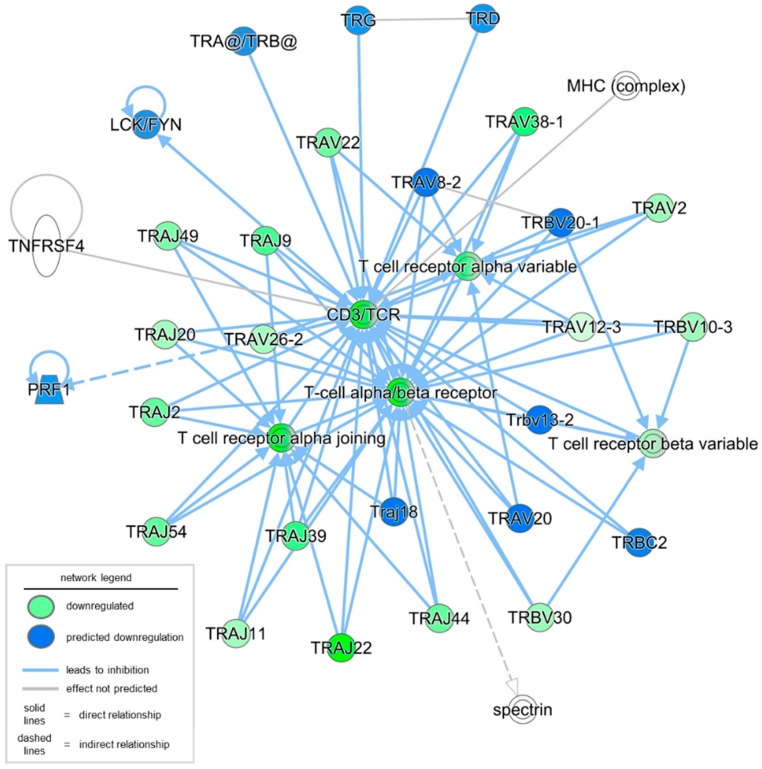
Network analysis of immunological disease and cellular compromise functions was conducted using IPA. A total of 16 focus molecules (in green) were downregulated in severe PU as compared to grade II PU. The intensity of the color represents the magnitude of molecules being down-regulated. An additional 19 molecules and relationships were also predicted; the majority of them were predicted to be downregulated (marked in blue).

To better understand the observed and predicted effector molecules involved in the defective T cell signaling in severe PU, a network analysis of immunological disease and cellular compromise functions was performed. The resulting network yielded a score of 27 with 16 downregulated genes and their fold changes in severe PU (Figure 2, Table 2). This included T cell receptor isoforms of variable (e.g., TRAVs/TRBVs), constant (e.g., TRBCs), and joining (e.g., TRAJs) segments (Figure 2, Table 2). Based on the expression levels of the above 16 focus molecules, an additional 19 molecules and relationships were predicted through IPA analysis (Figure 2, Table 2). In addition to predicted genes such as perforin 1 and T cell receptor-related genes, these included groups and complexes such as CD3/TCR, LCK/FYN, MHC, T cell receptors, TRA/TRB, and spectrin (Figure 2, Table 2). Disease and functional annotation of these 35 (16 observed and 19 predicted) molecules and relationships with respect to T lymphocyte function predicted their association with quantity, morphology, activation, and lack of T lymphocytes in severe PU subjects (Table 3).

**Figure 3:**
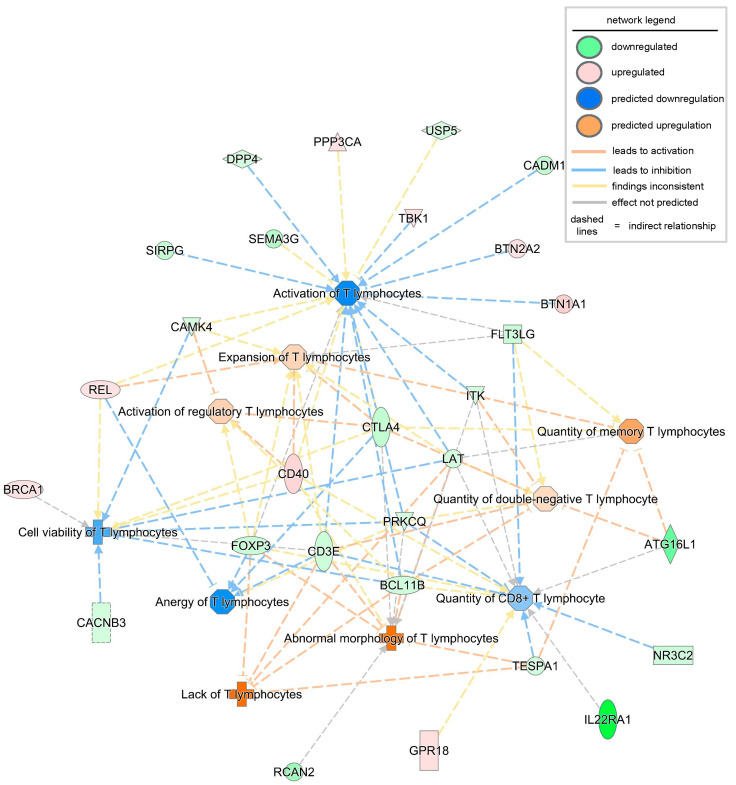
Disease or biofunction analysis related to T lymphocytes using IPA based on the observed and predicted differentially expressed genes and complexes in severe PU. The intensity of the color represents the magnitude of genes or pathways being up- or down-regulated.

Next, to understand the functional readouts of the observed DEGs and predicted complexes related to T lymphocyte abundance, function, and signaling, a disease and function annotation analysis was conducted using the IPA tool. All disease or function terms, including T lymphocytes with a significant *p*-value and an activation z-score greater than 0, were included. The first functional term that was significantly increased (both at *p*-value and activation z-score) in severe PU was the lack of T lymphocytes (Figure 3, Table 4). This function included five DEGs, i.e., *BCL11B*, *CD3E*, *FOXP3*, *LAT*, and *TESPA1*. FOXP3 is a master transcription factor for regulatory T cells (Tregs), and its expression is likely reduced or its function impaired in chronic inflammatory conditions such as PUs [35]. Additionally, BCL11B binds to the regulatory region of genes crucial for the Treg program, including FOXP3, to control Treg cell stability and function [36]. The combined deficiency of FOXP3 and BCL11B, as observed in severe PUs, explains the dysfunction of Treg cells and the development of an inflammatory phenotype [36]. These molecules, in addition to other DEGs such as *DPP4*, *CADM1*, *USP5*, and *FLT3LG*, resulted in decreased T lymphocyte activation and decreased quantity of T lymphocytes in severe PU (Figure 3, Table 4). Interestingly, when severe pressure ulcers were segregated by diabetic status, the lack of the T lymphocytes pathway was also significantly downregulated in diabetics, along with decreased recruitment and interactions of T lymphocytes (Figure S1). Combined diminished levels of cytokines such as *CCL20* [37], *CXCL9* [38], *CXCL10* [38], *CCL2* [39], and CXCL8 [40] indicated deficits in Treg recruitment and communication in diabetic compared to non-diabetic severe PU cases (Figure S1).

**Figure 4:**
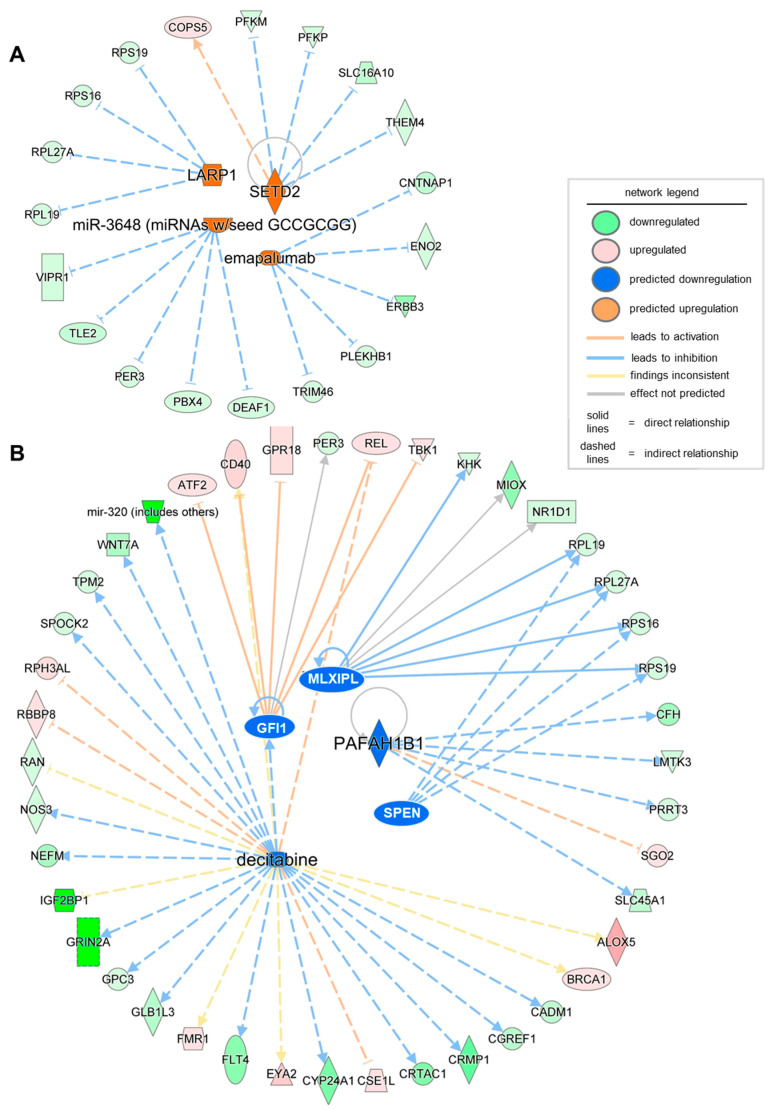
Network of significant (activation z-score > ±2, *p*-value < 0.05) activated (**A**) and inhibited (**B**) upstream regulators obtained using the upstream analysis function of IPA. The intensity of the color represents the magnitude of molecules or upstream regulators being up- or down-regulated.

Finally, to design future therapeutics based on the significant DEGs in severe PU, it is essential to identify the activated and inhibited upstream regulators, including drugs, transcription/translation regulators, enzymes, or microRNAs. Using the upstream analysis function in IPA, a list of significant upstream regulators (activation z-score > ±2, *p*-value < 0.05) was obtained, including both activated and inhibited regulators (Table 5). Targets of the translation regulator LARP1, enzyme SETD2, microRNA miR-3648, and the biological drug emapalumab were found to be differentially expressed in severe PU (Figure 4, Table 5). On the contrary, transcriptional regulators GFU1, SPEN, and MLXIPL, the enzyme PAFAH1B1, and the chemical drug decitabine, were found to be activated based on their target genes in the severe PU subjects (Figure 4, Table 5). Among the abovementioned activated and inhibited upstream regulators, decitabine was found to target the most significant number of genes in the datasets, and 19 of 26 genes showed measurement direction consistent with decitabine inhibition.

**Figure 5:**
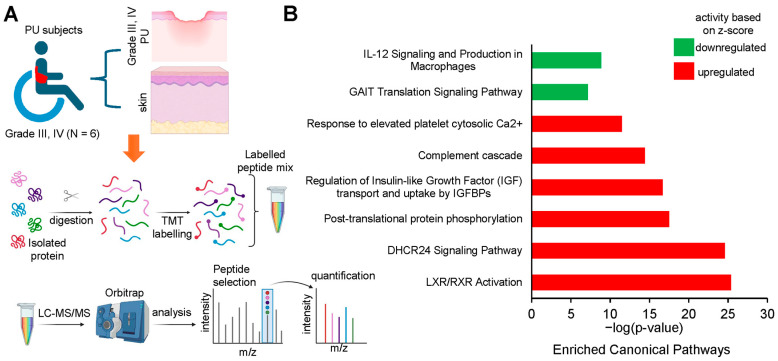
(**A**) Schematic diagram showing the layout of the proteomic study conducted by Baldan-Martin et al., 2020 [27]. Created in BioRender. Differentially expressed proteins identified by tandem mass tag (TMT) labeling in severe PU (grade III–IV) pressure ulcer tissue (*n* = 6) and control tissue (*n* = 6). (**B**) Bar chart created from IPA software output representing the significant canonical pathways using a strict criterion [activation z-score > ±3; −log(*p*-value) > 7].

**Figure 6:**
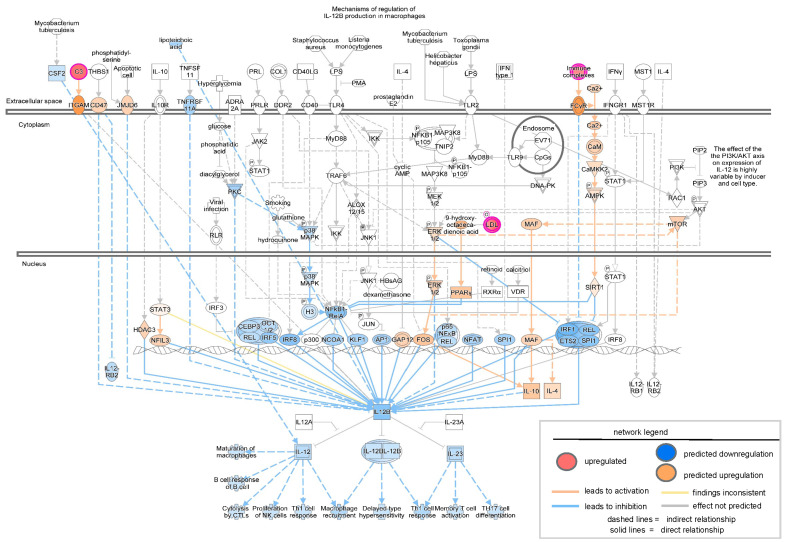
Network analysis of downregulated IL-12 signaling and production in macrophages as revealed by IPA analysis in the proteomic study conducted by Baldan-Martin et al., 2020 [27]. The intensity of the color represents the magnitude of molecules or complexes being up- or down-regulated.

**Figure 7:**
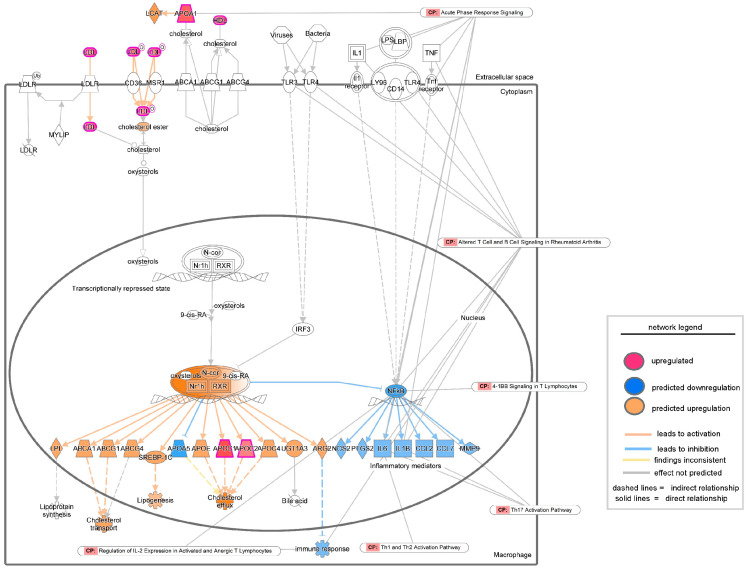
Network analysis of upregulated LXR/RXR activation signaling as revealed by IPA analysis in the proteomic study conducted by Baldan-Martin et al., 2020 [27]. The intensity of the color represents the magnitude of molecules or processes being up- or down-regulated. CP = Canonical Pathways.

To validate the findings from transcriptomic analysis of severe PU patients, tissue proteomics data generated by Baldan-Martin et al. [27] were analyzed using IPA. A total of six grade III/IV severe PU tissues were analyzed using tandem mass tags (TMT) followed by liquid chromatography–tandem mass spectrometry (LC-MS/MS) (Figure 5A). Adjacent healthy tissues with a typical layered structure, well-packed collagen matrix, and well-formed vasculature, were used as controls (Figure 5A). Out of a total of 4504 proteins, 76 showed significant abundance differences, as presented in Table S2, and were used for IPA analysis. Canonical pathways enriched with the differentially abundant proteins using a strict criterion [activation z-score > ±3; −log(*p*-value) > 7] yielded IL-12 signaling and production in macrophages to be the most significant pathway downregulated in severe pressure ulcer tissue as compared to control (Figure 5B). The proteins involved in the dataset related to this pathway were APOA1, APOA2, APOB, APOC1, APOC2, APOC3, C3, IGHG1, SAA4, and SERPIN. The biological signaling networks involved in this pathway were then evaluated using IPA to understand the molecular processes affected (Figure 6). T lymphocyte functional readouts, such as Th1 cell response, memory T cell activation, and TH17 cell differentiation, were found to be downregulated by reduced levels of IL12 B and IL23 (Figure 6). On the other hand, the topmost significant upregulated pathway in severe PU tissue was LXR/RXR activation [based on −log(*p*-value)] (Figure 6), which was composed of APOA1, APOA2, APOB, APOC1, APOC2, APOC3, C3, C4B, GC, HPX, ITIH4, KNG1, PON3, SAA4, SERPIN, TF, and TTR proteins. This pathway remains involved in (i) acute phase response, (ii) results in altered T cell and B cell signaling, (iii) 4-1 BB signaling in T lymphocytes, (iv) regulation of IL-2 expression in activated and anergic T lymphocytes, (v) Th1 and Th2 activation pathway, and (vi) Th17 activation pathways in macrophages (Figure 7). Taken together, an independent tissue-level proteomic dataset validated the findings of the systemic transcriptomics data, indicating the compromised immune response in severe PU subjects, predominantly due to deficient T lymphocyte activation and function. However, being purely computational and descriptive, the scope of this work is limited to cataloging molecules and complexes that are differentially expressed in severe PU compared to grade II PU. Additionally, integrating transcriptomic and proteomic data from two different studies may introduce additional bias. Future experimental validations using tandem spatial transcriptomics and proteomics approaches in the serial sections of the PU wound-edge tissue will provide an in-depth understanding of the observed findings.

## 4. Discussion

Pressure ulcers (PUs) represent a systemic and multifactorial disease that often results in disability and fatal infections [41]. PUs usually affect older patients, being observed in more than 80% of hospitalized individuals within the first five days of the inpatient hospital stay [5]. Since many early-stage PUs can be prevented or reversed, the availability of physical, molecular, or biochemical biomarkers that may predict disease severity and progression could provide significant benefits to both patients and the healthcare system [26,27,42]. To that end, several blood- or tissue-based biomarkers are being investigated using multi-omics (proteomic and transcriptomic) approaches. The findings of the current study reveal a combined systemic and local alteration in the inflammatory and immune status, mainly affecting T cell function and communication as a molecular signature that may help distinguish PU severity. The observed findings may also apply to other chronic wounds, such as diabetic foot ulcers (DFUs) or severe burn wounds, where T cell composition and number regulate inflammation and the healing response [43,44,45,46]. For example, a study demonstrating the single-cell transcriptomic landscape of DFUs showed that, compared with nonhealing DFUs, healing DFUs contain higher proportions of naive and early-differentiated progenitor T lymphocytes, which activate various T cell subsets [43]. Moreover, accumulation of Foxp3^+^ Treg cells using stem cells at the diabetic wound site creates a regenerative immune microenvironment and accelerates healing [47]. As effector T cell accumulation and TCR repertoire diversity reduction appear to precede the development of foot ulcers [48], the current findings may guide the development of new T cell-targeted therapies to promote the healing of severe PU [49].

At the RNA level, enrichment of differentially expressed genes indicated T cell dysfunction and a compromised broader T cell regulatory network in severe PU subjects. For example, downregulation of *CD3E* expression, which often results in reduced IL-2 expression in anergic T lymphocytes, suggested insufficient TCR activation. Moreover, reduced expression of the downstream effector *CTLA4* in severe PU subjects suggested a combined deficiency in CD80/86- and CD28-mediated T cell activation and survival.

A protein-based study further validated this finding, predicting that T lymphocyte functional readouts such as Th1 cell responses, memory T cell activation, and TH17 cell differentiation would be downregulated. Indeed, systemic inflammation, often observed in chronic wound tissue, such as in diabetics, decreases the abundance and migration of Tregs while promoting the infiltration of inflammatory Th17 cells [50,51]. Additionally, a subset of Tregs can promote the formation of new blood vessels in diabetic mice following ischemic injury. Furthermore, exogenous Tregs decrease neutrophil and cytotoxic T cell accumulation as well as IFN-γ production in damaged tissues, further reducing inflammation [52].

Our transcriptomics and proteomics data-mining approach supported the current literature that in severe PU, although inflammation is persistent, it is ineffective in combating infection and healing wounds due to the prevalence of low-responsive T cells [53,54]. In line with this observation, an independent study reported that T cells from PUs enriched with major histocompatibility complex II^+^ keratinocytes (MHC II^+^ KC) produced fewer inflammatory cytokines [24]. Furthermore, these MHC II^+^ KC may directly interact with T cells through antigen presentation and reduce T cell recruitment and activation, as represented by reduced cytokine concentrations such as IFN-γ, IL-9, IL-21, GM-CSF, CXCL1, CXCL8, CCL4, and CXCL12 [24]. This finding supported the hypothesis that cell–cell communication between MHC II^+^ KCs and T cells might interfere with T cell function in PU. Similar findings have been reported in previous studies showing that KC–T cell interactions can lead to T cell anergy or tolerance [55,56]. In addition, epidermal T cells isolated from human chronic wounds are less responsive to stimulation than T cells from acute wounds [54]. Moreover, the TCR-induced T cell proliferation decreased from 7.6% to 1.2% (P = 0.03) by coculturing with KCs pretreated with MHC II^+^ KC-derived wound fluid. These results suggested that KCs might function as atypical antigen-presenting cells and exert an inhibitory effect on T cell activation in PU wound edges.

## 5. Conclusions

Significant efforts are being made to understand the mechanisms underlying the development of severe PU in patients. The present study adopted a multi-omic approach, integrating systemic transcriptomics and tissue-level proteomics to obtain a holistic understanding of cellular mechanisms at the molecular level in PU subjects. The findings reveal a combined systemic and local alteration in the inflammatory and immune status predominantly affecting T cell function and communication as a molecular signature that may help distinguish PU severity and inform the development of a cell-based therapy for regenerative medicine.

## Figures and Tables

**Table 1:** List of canonical pathways and associated molecules enriched in severe PU (grade III–IV) as compared to grade II PU.

Ingenuity Canonical Pathways	−log (*p*-Value)	z-Score	Molecules
ICOS-ICOSL Signaling in T Helper Cells	5.54	−0.816	*CD3E*, *CD40*, *ITK*, *LAT*, *PLCG1*, *PLEKHA2*, *PPP3CA*, *PRKCQ*, *REL*, *TRAJ11*, *TRAJ2*, *TRAJ20*, *TRAJ22*, *TRAJ39*, *TRAJ44*, *TRAJ49*, *TRAJ54*, *TRAJ9*, *TRAV12-3*, *TRAV2*, *TRAV22*, *TRAV26-2*, *TRAV38-1*, *TRBV10-3*, *TRBV30*
CD28 Signaling in T Helper Cells	4.9	−0.816	*CD3E*, *CTLA4*, *ITK*, *LAT*, *PLCG1*, *PPP3CA*, *PRKCQ*, *REL*, *TRAJ11*, *TRAJ2*, *TRAJ20*, *TRAJ22*, *TRAJ39*, *TRAJ44*, *TRAJ49*, *TRAJ54*, *TRAJ9*, *TRAV12-3*, *TRAV2*, *TRAV22*, *TRAV26-2*, *TRAV38-1*, *TRBV10-3*, *TRBV30*
PKCθ Signaling in T Lymphocytes	4.87	−1.342	*CACNA1F*, *CACNA1H*, *CACNB3*, *CD3E*, *LAT*, *PLCG1*, *PPP3CA*, *PRKCQ*, *REL*, *TRAJ11*, *TRAJ2*, *TRAJ20*, *TRAJ22*, *TRAJ39*, *TRAJ44*, *TRAJ49*, *TRAJ54*, *TRAJ9*, *TRAV12-3*, *TRAV2*, *TRAV22*, *TRAV26-2*, *TRAV38-1*, *TRBV10-3*, *TRBV30*
Regulation of IL-2 Expression in Activated and Anergic T Lymphocytes	4.72	−3.838	*CD3E*, *LAT*, *PLCG1*, *PPP3CA*, *PRKCQ*, *REL*, *TRAJ11*, *TRAJ2*, *TRAJ20*, *TRAJ22*, *TRAJ39*, *TRAJ44*, *TRAJ49*, *TRAJ54*, *TRAJ9*, *TRAV12-3*, *TRAV2*, *TRAV22*, *TRAV26-2*, *TRAV38-1*, *TRBV10-3*, *TRBV30*
Role of NFAT in Cardiac Hypertrophy	4.38	−1.508	*CACNA1F*, *CACNA1H*, *CACNB3*, *CAMK4*, *CSNK1A1*, *HDAC2*, *IL11*, *MEF2B*, *NOTUM*, *PLCG1*, *PPP3CA*, *PRKCQ*, *RCAN2*, *SHF*
Calcium-induced T Lymphocyte Apoptosis	4.25	−1	*CD3E*, *HDAC2*, *PLCG1*, *PPP3CA*, *PRKCQ*, *TRAJ11*, *TRAJ2*, *TRAJ20*, *TRAJ22*, *TRAJ39*, *TRAJ44*, *TRAJ49*, *TRAJ54*, *TRAJ9*, *TRAV12-3*, *TRAV2*, *TRAV22*, *TRAV26-2*, *TRAV38-1*, *TRBV10-3*, *TRBV30*
T Cell Receptor Signaling	4.12	−3.4	*ATF2*, *CD3E*, *CTLA4*, *ITK*, *LAT*, *PLCG1*, *PPP3CA*, *PRKCQ*, *REL*, *TRAJ11*, *TRAJ2*, *TRAJ20*, *TRAJ22*, *TRAJ39*, *TRAJ44*, *TRAJ49*, *TRAJ54*, *TRAJ9*, *TRAV12-3*, *TRAV2*, *TRAV22*, *TRAV26-2*, *TRAV38-1*, *TRBV10-3*, *TRBV30*
Chaperone-Mediated Autophagy Signaling Pathway	3.96	−3.4	*CD3E*, *EIF2AK3*, *HDAC2*, *MMP27*, *NOS3*, *PPP3CA*, *PSMB3*, *RILP*, *TBK1*, *TRAJ11*, *TRAJ2*, *TRAJ20*, *TRAJ22*, *TRAJ39*, *TRAJ44*, *TRAJ49*, *TRAJ54*, *TRAJ9*, *TRAV12-3*, *TRAV2*, *TRAV22*, *TRAV26-2*, *TRAV38-1*, *TRBV10-3*, *TRBV30*
CTLA4 Signaling in Cytotoxic T Lymphocytes	3.83	3.962	*CD3E*, *CTLA4*, *FOXP3*, *ITK*, *LAT*, *PLCG1*, *PPP2R2A*, *PRKCQ*, *TRAJ11*, *TRAJ2*, *TRAJ20*, *TRAJ22*, *TRAJ39*, *TRAJ44*, *TRAJ49*, *TRAJ54*, *TRAJ9*, *TRAV12-3*, *TRAV2*, *TRAV22*, *TRAV26-2*, *TRAV38-1*, *TRBV10-3*, *TRBV30*
G Protein Signaling Mediated by Tubby	3.74	−4.472	*CD3E*, *ITK*, *LAT*, *PLCG1*, *TRAJ11*, *TRAJ2*, *TRAJ20*, *TRAJ22*, *TRAJ39*, *TRAJ44*, *TRAJ49*, *TRAJ54*, *TRAJ9*, *TRAV12-3*, *TRAV2*, *TRAV22*, *TRAV26-2*, *TRAV38-1*, *TRBV10-3*, *TRBV30*
NFKBIE Signaling Pathway	3.54	−3.441	*CD3E*, *CD40*, *REL*, *TRAJ11*, *TRAJ2*, *TRAJ20*, *TRAJ22*, *TRAJ39*, *TRAJ44*, *TRAJ49*, *TRAJ54*, *TRAJ9*, *TRAV12-3*, *TRAV2*, *TRAV22*, *TRAV26-2*, *TRAV38-1*, *TRBV10-3*, *TRBV30*
Calcium Signaling	3.42	−0.707	*ATF2*, *CACNA1F*, *CACNA1H*, *CACNB3*, *CAMK4*, *GRIN2A*, *HDAC2*, *MEF2B*, *MYL3*, *PPP3CA*, *RCAN2*, *TPM2*
NUR77 Signaling in T Lymphocytes	3.18	−1	*CD3E*, *HDAC2*, *PPP3CA*, *PRKCQ*, *TRAJ11*, *TRAJ2*, *TRAJ20*, *TRAJ22*, *TRAJ39*, *TRAJ44*, *TRAJ49*, *TRAJ54*, *TRAJ9*, *TRAV12-3*, *TRAV2*, *TRAV22*, *TRAV26-2*, *TRAV38-1*, *TRBV10-3*, *TRBV30*
Lipid Antigen Presentation by CD1	3.08	−4.123	*CD3E*, *TRAJ11*, *TRAJ2*, *TRAJ20*, *TRAJ22*, *TRAJ39*, *TRAJ44*, *TRAJ49*, *TRAJ54*, *TRAJ9*, *TRAV12-3*, *TRAV2*, *TRAV22*, *TRAV26-2*, *TRAV38-1*, *TRBV10-3*, *TRBV30*
Corticotropin Releasing Hormone Signaling	2.86	−1	*ATF2*, *CACNA1F*, *CACNA1H*, *CACNB3*, *CAMK4*, *MEF2B*, *NOS3*, *PLCG1*, *PRKCQ*
IL-4 Signaling	2.65	−3.578	*ATF2*, *CD3E*, *COL6A1*, *GAB1*, *TRAJ11*, *TRAJ2*, *TRAJ20*, *TRAJ22*, *TRAJ39*, *TRAJ44*, *TRAJ49*, *TRAJ54*, *TRAJ9*, *TRAV12-3*, *TRAV2*, *TRAV22*, *TRAV26-2*, *TRAV38-1*, *TRBV10-3*, *TRBV30*
Glutaminergic Receptor Signaling Pathway (Enhanced)	2.39	−0.832	*ATF2*, *CACNA1F*, *CACNA1H*, *CACNB3*, *CAMK4*, *DGKA*, *FMR1*, *GRIN2A*, *NOTUM*, *PLCG1*, *PPP3CA*, *PRKCQ*, *SLC38A2*
Dilated Cardiomyopathy Signaling Pathway	2.36	−2.236	*BAG3*, *CACNA1F*, *CACNA1H*, *CACNB3*, *CAMK4*, *GAB1*, *MYL3*, *SGCD*
IL-15 Production	2.31	−1.633	*DYRK4*, *ERBB3*, *FLT3LG*, *FLT4*, *ITK*, *LMTK3*, *REL*
Synaptic Long-Term Potentiation	2.27	−0.816	*ATF2*, *CAMK4*, *GRIN2A*, *NOTUM*, *PLCG1*, *PPP3CA*, *PRKCQ*
Zn Homeostasis Signaling Pathway	2.17	2.921	*ATF2*, *CD3E*, *CYSLTR2*, *GPR18*, *GPRC5C*, *GRIN2A*, *HTR6*, *MMP27*, *PPP3CA*, *PRKCQ*, *RHO*, *SELENOW*, *SLC52A1*, *TRAJ11*, *TRAJ2*, *TRAJ20*, *TRAJ22*, *TRAJ39*, *TRAJ44*, *TRAJ49*, *TRAJ54*, *TRAJ9*, *TRAV12-3*, *TRAV2*, *TRAV22*, *TRAV26-2*, *TRAV38-1*, *TRBV10-3*, *TRBV30*, *VIPR1*
Immunoregulatory Interactions between a Lymphoid and a Non-Lymphoid Cell	2.08	−3.441	*CD3E*, *CD40*, *FCGR2B*, *TRAJ11*, *TRAJ2*, *TRAJ20*, *TRAJ22*, *TRAJ39*, *TRAJ44*, *TRAJ49*, *TRAJ54*, *TRAJ9*, *TRAV12-3*, *TRAV2*, *TRAV22*, *TRAV26-2*, *TRAV38-1*, *TRBV10-3*, *TRBV30*
Inositol Phosphate Metabolism	2.05	−1	*INPP4B*, *MIOX*, *PLCG1*, *PPIP5K2*
CREB Signaling in Neurons	2.01	−1.698	*ATF2*, *CACNA1F*, *CACNA1H*, *CACNB3*, *CAMK4*, *CYSLTR2*, *FLT4*, *GPR18*, *GPRC5C*, *GRIN2A*, *HTR6*, *NOTUM*, *PLCG1*, *POLR2F*, *PRKCQ*, *RHO*, *SHF*, *SLC52A1*, *VIPR1*

**Table 2:** List of 35 focus molecules and complexes (observed and predicted) involved in immunological disease and cellular compromise function as demonstrated in Figure 2. These molecules and complexes were divided into three categories based on the fold change and activation status. A. Downregulated in the dataset: this included 16 genes, 3 groups, and 2 complexes. B. Predicted to be downregulated in the dataset: this included 9 genes, 1 group, and 1 complex. C. No activity pattern available in the dataset: this included 1 gene, 1 group, and 1 complex.

Symbol	Entrez Gene Name	log2 Fold Change	Type of Molecule
A. Genes, groups, and complexes downregulated in the dataset			
*TRAJ22*	T cell receptor alpha joining 22	−3.628619755	Gene
*TRAV38-1*	T cell receptor alpha variable 38-1	−2.072440415	Gene
*TRAJ39*	T cell receptor alpha joining 39	−1.823949277	Gene
*TRAJ9*	T cell receptor alpha joining 9	−1.709212257	Gene
*TRAJ2*	T cell receptor alpha joining 2 (non-functional)	−1.588144944	Gene
*TRAJ54*	T cell receptor alpha joining 54	−1.582453079	Gene
*TRAJ44*	T cell receptor alpha joining 44	−1.567723326	Gene
*TRAV22*	T cell receptor alpha variable 22	−1.497050695	Gene
*TRAJ49*	T cell receptor alpha joining 49	−1.378685736	Gene
*TRAV2*	T cell receptor alpha variable 2	−1.156064019	Gene
*TRBV30*	T cell receptor beta variable 30	−1.150917618	Gene
*TRBV10-3*	T cell receptor beta variable 10-3	−1.131684815	Gene
*TRAJ11*	T cell receptor alpha joining 11	−1.095500661	Gene
*TRAJ20*	T cell receptor alpha joining 20	−1.054293781	Gene
*TRAV26-2*	T cell receptor alpha variable 26-2	−1.021698834	Gene
*TRAV12-3*	T cell receptor alpha variable 12-3	−0.605153066	Gene
T cell receptor alpha joining		N/A	Group
T cell receptor alpha variable		N/A	Group
T cell receptor beta variable		N/A	Group
CD3/TCR		N/A	Complex
T cell alpha/beta receptor		N/A	Complex
B. Genes, groups, and complexes predicted to be downregulated in the dataset			
PRF1	perforin 1	predicted downregulated	Gene
Traj18	T cell receptor alpha joining 18	predicted downregulated	Gene
TRAV20	T cell receptor alpha variable 20	predicted downregulated	Gene
TRAV8-2	T cell receptor alpha variable 8-2	predicted downregulated	Gene
TRBC2	T cell receptor beta constant 2	predicted downregulated	Gene
Trbv13-2	T cell receptor beta, variable 13-2	predicted downregulated	Gene
TRBV20-1	T cell receptor beta variable 20-1	predicted downregulated	Gene
TRD	T cell receptor delta locus	predicted downregulated	Gene
TRG	T cell receptor gamma locus	predicted downregulated	Gene
LCK/FYN		predicted downregulated	Group
TRA@/TRB@		predicted downregulated	Complex
C. Genes, groups, and complexes with no activity pattern available			
TNFRSF4	TNF receptor superfamily member 4	no activity reported	Gene
Spectrin		no activity reported	Group
MHC (complex)		no activity reported	Complex

**Table 3:** List of predicted molecules in Figure 2 and enriched T lymphocyte function annotation.

Diseases or Functions Annotation	*p*-Value	Molecules
Quantity of T lymphocytes	0.0298	PRF1, TNFRSF4, Traj18, Trbv13-2, TRG
Abnormal morphology of T lymphocytes	0.00233	TNFRSF4, Traj18, TRG
Deletion of T lymphocytes	0.0000256	PRF1, TNFRSF4, Trbv13-2
Quantity of activated T lymphocytes	0.000862	PRF1, Traj18
Lack of T lymphocytes	0.000973	Traj18, TRG
Activation of CD8+ T lymphocytes	0.0119	PRF1, TRG
Activation-induced cell death of T lymphocytes	0.00611	PRF1, TNFRSF4
Lack of natural killer T lymphocytes	0.00795	Traj18
Lack of gamma-delta T lymphocytes	0.0118	TRG
Deletion of naive T lymphocytes	0.00136	PRF1
Number of activated T lymphocytes	0.0412	PRF1
Expansion of effector T lymphocytes	0.0229	TNFRSF4
Elimination of T lymphocytes	0.0147	PRF1
Conversion of T lymphocytes	0.0488	TNFRSF4
Cytotoxicity of CD8+ T lymphocytes	0.0269	PRF1
Apoptosis of naive T lymphocytes	0.0122	PRF1
Suppression of effector T lymphocytes	0.0105	TNFRSF4
Killing by effector T lymphocytes	0.00136	PRF1
Differentiation of natural killer T lymphocytes	0.0303	Traj18
Deletion of memory T lymphocytes	0.00136	PRF1
Malignancy of natural killer T lymphocytes	0.00136	PRF1
Activation-induced cell death of CD8+ T lymphocytes	0.0118	PRF1

**Table 4:** List of significantly enriched diseases or biofunctions related to T lymphocytes in severe PU as shown in Figure 3.

Diseases or Functions Annotation	*p*-Value	Predicted Activation State	Activation z-Score	Molecules	#Molecules
Lack of T lymphocytes	0.0000984	Increased	2.183	*BCL11B*, *CD3E*, *FOXP3*, *LAT*, *TESPA1*	5
Abnormal morphology of T lymphocytes	0.000229		1.823	*BCL11B*, *CD3E*, *CD40*, *CTLA4*, *FOXP3*, *ITK*, *LAT*, *PRKCQ*, *RCAN2*, *TESPA1*	10
Quantity of memory T lymphocytes	0.0162		0.853	*ATG16L1*, *CTLA4*, *FLT3LG*, *ITK*, *LAT*, *TESPA1*	6
Activation of regulatory T lymphocytes	0.000782		0.391	*BCL11B*, *CAMK4*, *CD40*, *CTLA4*, *FOXP3*	5
Expansion of T lymphocytes	0.0101		0.355	*CAMK4*, *CD3E*, *CD40*, *CTLA4*, *FLT3LG*, *FOXP3*, *ITK*, *LAT*, *REL*	9
Quantity of double-negative T lymphocytes	0.000234		0.314	*ATG16L1*, *BCL11B*, *CD3E*, *CTLA4*, *FLT3LG*, *ITK*, *LAT*, *PRKCQ*	8
Quantity of CD8+ T lymphocytes	0.00378		−0.513	*ATG16L1*, *BCL11B*, *CD3E*, *CD40*, *FLT3LG*, *FOXP3*, *GPR18*, *IL22RA1*, *ITK*, *LAT*, *NR3C2*, *PRKCQ*, *TESPA1*	13
Cell viability of T lymphocytes	0.00027		−0.787	*BCL11B*, *BRCA1*, *CACNB3*, *CAMK4*, *CD3E*, *CD40*, *CTLA4*, *LAT*, *PRKCQ*, *REL*	10
Anergy of T lymphocytes	0.000222		−1.109	*CD3E*, *CTLA4*, *FOXP3*, *PRKCQ*, *REL*	5
Activation of T lymphocytes	0.00502		−1.23	*BCL11B*, *BTN1A1*, *BTN2A2*, *CADM1*, *CAMK4*, *CD3E*, *CD40*, *CTLA4*, *DPP4*, *FLT3LG*, *FOXP3*, *ITK*, *LAT*, *PPP3CA*, *PRKCQ*, *REL*, *SEMA3G*, *SIRPG*, *TBK1*, *USP5*	20

**Table 5:** List of significant (activation z-score > ±2, *p*-value < 0.05) activated and inhibited upstream regulators obtained using the upstream analysis function of IPA.

Upstream Regulator	Molecule Type	Predicted Activation State	Activation z-Score	*p*-Value of Overlap	Target Molecules in Dataset
LARP1	translation regulator	Activated	2	0.045	*RPL19*, *RPL27A*, *RPS16*, *RPS19*
SETD2	enzyme	Activated	2.236	0.0178	*COPS5*, *PFKM*, *PFKP*, *SLC16A10*, *THEM4*
miR-3648 (miRNAs w/seed GCCGCGG)	mature microRNA	Activated	2.236	0.0299	*DEAF1*, *PBX4*, *PER3*, *TLE2*, *VIPR1*
emapalumab	biologic drug	Activated	2.236	0.000132	*CNTNAP1*, *ENO2*, *ERBB3*, *PLEKHB1*, *TRIM46*
GFI1	transcription regulator	Inhibited	−2.236	0.041	*ATF2*, *CD40*, *GPR18*, *PER3*, *REL*, *TBK1*
PAFAH1B1	enzyme	Inhibited	−2.236	0.0144	*CFH*, *LMTK3*, *PRRT3*, *SGO2*, *SLC45A1*
MLXIPL	transcription regulator	Inhibited	−2.177	0.0122	*KHK*, *MIOX*, *NR1D1*, *RPL19*, *RPL27A*, *RPS16*, *RPS19*
decitabine	chemical drug	Inhibited	−2.162	0.0157	*ALOX5*, *BRCA1*, *CADM1*, *CD40*, *CGREF1*, *CRMP1*, *CRTAC1*, *CSE1L*, *CYP24A1*, *EYA2*
SPEN	transcription regulator	Inhibited	−2	0.0172	*RPL19*, *RPL27A*, *RPS16*, *RPS19*

## Data Availability

The bioinformatic analysis conducted in this manuscript is derived from the normalized RNA sequencing data available in GEO database under accession numbers: GEO Submission (GSE230161) and the differentially expressed proteins identified by tandem mass tag (TMT) labeling presented by Baldan-Martin et al., 2020 (Adv Wound Care) [27]. The original contributions of bioinformatics data mining presented in this study are included in the article/Supplementary Material. Further inquiries can be directed to the corresponding author.

## Supplementary Materials

The following supporting information can be downloaded at: https://www.mdpi.com/article/10.3390/biom15121682/s1, Figure S1: Disease or biofunction analysis related to T lymphocytes using IPA based on the observed and predicted differential expressed genes and complexes in diabetic severe PU as compared to non-diabetic severe PUs; Table S1: Patient phenotypes and characteristics for which RNA sequencing data was downloaded from GEO database under accession number: GSE230161; Table S2: Differentially expressed proteins identified by tandem mass tag labeling in pressure ulcer tissue and control tissue reported by Baldan-Martin et al. Adv Wound Care (New Rochelle). 2020 [27].
