# Supplementary material for: Comprehensive Transcriptomic and Proteomic Analysis of Severe Pressure Ulcer Patients Identifies Molecular Signatures Associated with Impaired T Cell Function
Source: Biomolecules. 2025 Dec 2;15(12):1682. doi: 10.3390/biom15121682 (PMC12731076; doi:10.3390/biom15121682)
Supplement: Supplementary file 1 [file biomolecules-15-01682-s001.zip › biomolecules-4000204-supplementary.pptx]

## Slide 1
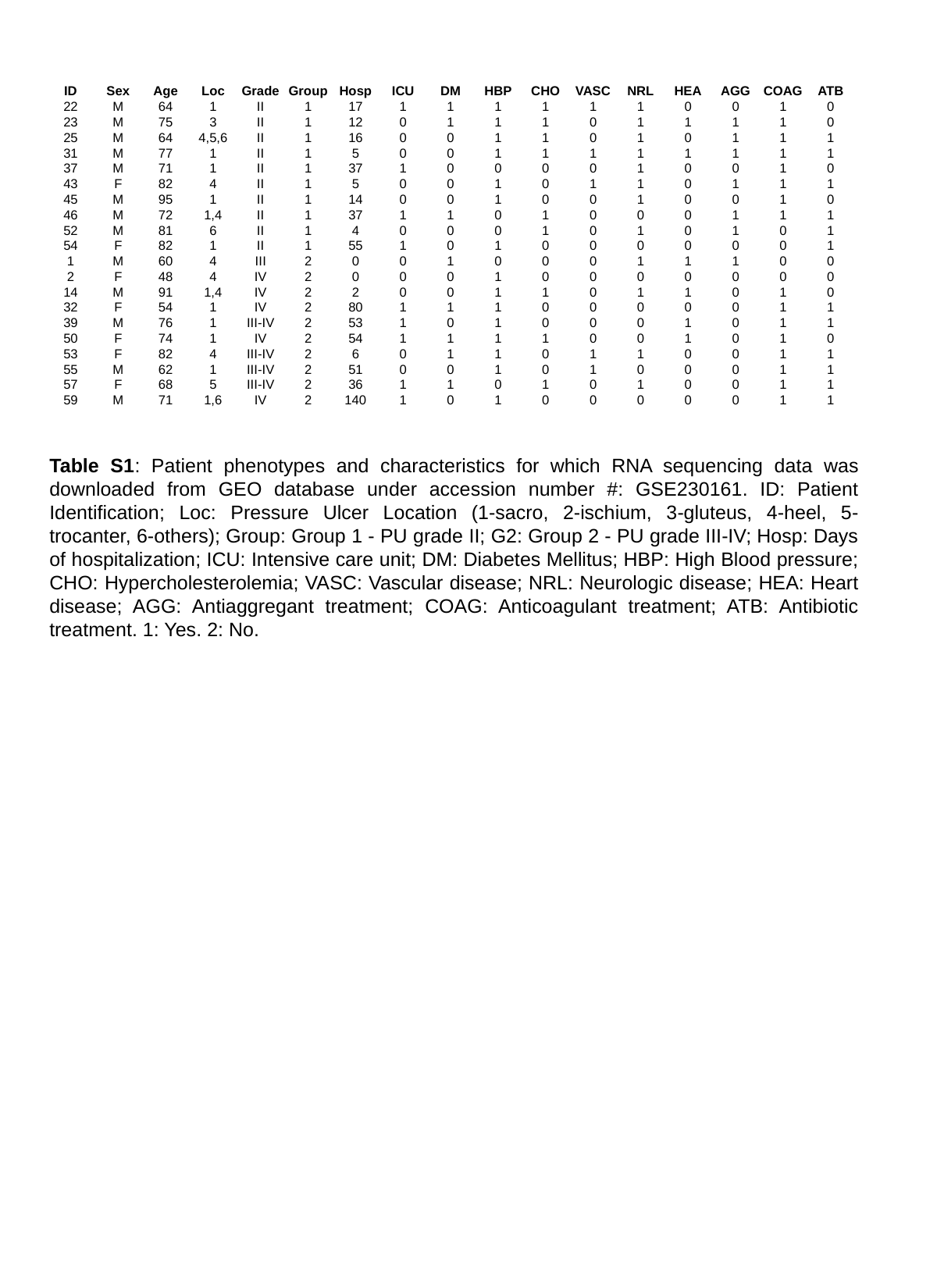

| ID | Sex | Age | Loc | Grade | Group | Hosp | ICU | DM | HBP | CHO | VASC | NRL | HEA | AGG | COAG | ATB |
| --- | --- | --- | --- | --- | --- | --- | --- | --- | --- | --- | --- | --- | --- | --- | --- | --- |
| 22 | M | 64 | 1 | II | 1 | 17 | 1 | 1 | 1 | 1 | 1 | 1 | 0 | 0 | 1 | 0 |
| 23 | M | 75 | 3 | II | 1 | 12 | 0 | 1 | 1 | 1 | 0 | 1 | 1 | 1 | 1 | 0 |
| 25 | M | 64 | 4,5,6 | II | 1 | 16 | 0 | 0 | 1 | 1 | 0 | 1 | 0 | 1 | 1 | 1 |
| 31 | M | 77 | 1 | II | 1 | 5 | 0 | 0 | 1 | 1 | 1 | 1 | 1 | 1 | 1 | 1 |
| 37 | M | 71 | 1 | II | 1 | 37 | 1 | 0 | 0 | 0 | 0 | 1 | 0 | 0 | 1 | 0 |
| 43 | F | 82 | 4 | II | 1 | 5 | 0 | 0 | 1 | 0 | 1 | 1 | 0 | 1 | 1 | 1 |
| 45 | M | 95 | 1 | II | 1 | 14 | 0 | 0 | 1 | 0 | 0 | 1 | 0 | 0 | 1 | 0 |
| 46 | M | 72 | 1,4 | II | 1 | 37 | 1 | 1 | 0 | 1 | 0 | 0 | 0 | 1 | 1 | 1 |
| 52 | M | 81 | 6 | II | 1 | 4 | 0 | 0 | 0 | 1 | 0 | 1 | 0 | 1 | 0 | 1 |
| 54 | F | 82 | 1 | II | 1 | 55 | 1 | 0 | 1 | 0 | 0 | 0 | 0 | 0 | 0 | 1 |
| 1 | M | 60 | 4 | III | 2 | 0 | 0 | 1 | 0 | 0 | 0 | 1 | 1 | 1 | 0 | 0 |
| 2 | F | 48 | 4 | IV | 2 | 0 | 0 | 0 | 1 | 0 | 0 | 0 | 0 | 0 | 0 | 0 |
| 14 | M | 91 | 1,4 | IV | 2 | 2 | 0 | 0 | 1 | 1 | 0 | 1 | 1 | 0 | 1 | 0 |
| 32 | F | 54 | 1 | IV | 2 | 80 | 1 | 1 | 1 | 0 | 0 | 0 | 0 | 0 | 1 | 1 |
| 39 | M | 76 | 1 | III-IV | 2 | 53 | 1 | 0 | 1 | 0 | 0 | 0 | 1 | 0 | 1 | 1 |
| 50 | F | 74 | 1 | IV | 2 | 54 | 1 | 1 | 1 | 1 | 0 | 0 | 1 | 0 | 1 | 0 |
| 53 | F | 82 | 4 | III-IV | 2 | 6 | 0 | 1 | 1 | 0 | 1 | 1 | 0 | 0 | 1 | 1 |
| 55 | M | 62 | 1 | III-IV | 2 | 51 | 0 | 0 | 1 | 0 | 1 | 0 | 0 | 0 | 1 | 1 |
| 57 | F | 68 | 5 | III-IV | 2 | 36 | 1 | 1 | 0 | 1 | 0 | 1 | 0 | 0 | 1 | 1 |
| 59 | M | 71 | 1,6 | IV | 2 | 140 | 1 | 0 | 1 | 0 | 0 | 0 | 0 | 0 | 1 | 1 |
Table S1: Patient phenotypes and characteristics for which RNA sequencing data was downloaded from GEO database under accession number #: GSE230161. ID: Patient Identification; Loc: Pressure Ulcer Location (1-sacro, 2-ischium, 3-gluteus, 4-heel, 5-trocanter, 6-others); Group: Group 1 - PU grade II; G2: Group 2 - PU grade III-IV; Hosp: Days of hospitalization; ICU: Intensive care unit; DM: Diabetes Mellitus; HBP: High Blood pressure; CHO: Hypercholesterolemia; VASC: Vascular disease; NRL: Neurologic disease; HEA: Heart disease; AGG: Antiaggregant treatment; COAG: Anticoagulant treatment; ATB: Antibiotic treatment. 1: Yes. 2: No.

## Slide 2
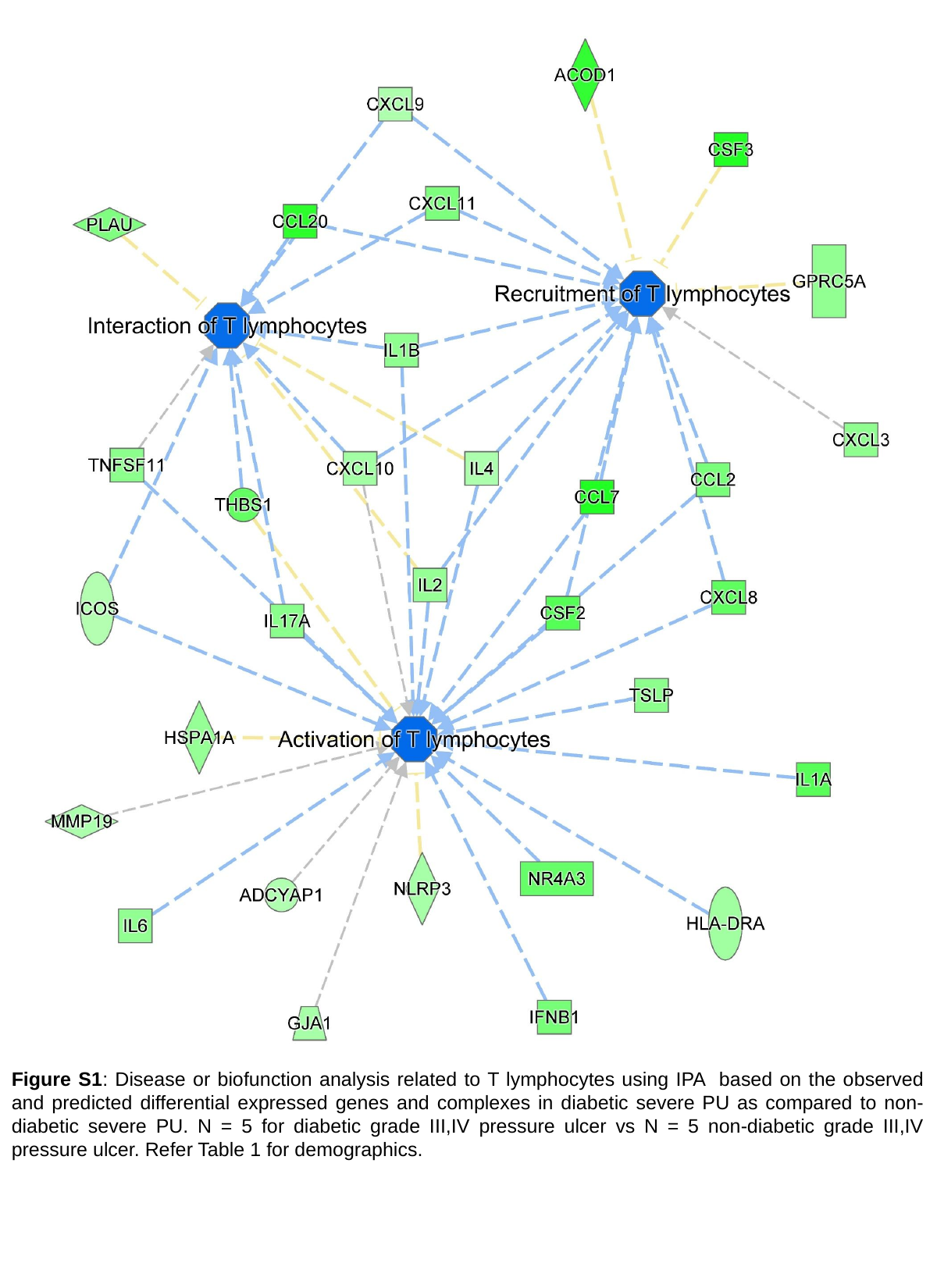

Figure S1: Disease or biofunction analysis related to T lymphocytes using IPA based on the observed and predicted differential expressed genes and complexes in diabetic severe PU as compared to non-diabetic severe PU. N = 5 for diabetic grade III,IV pressure ulcer vs N = 5 non-diabetic grade III,IV pressure ulcer. Refer Table 1 for demographics.

## Slide 3
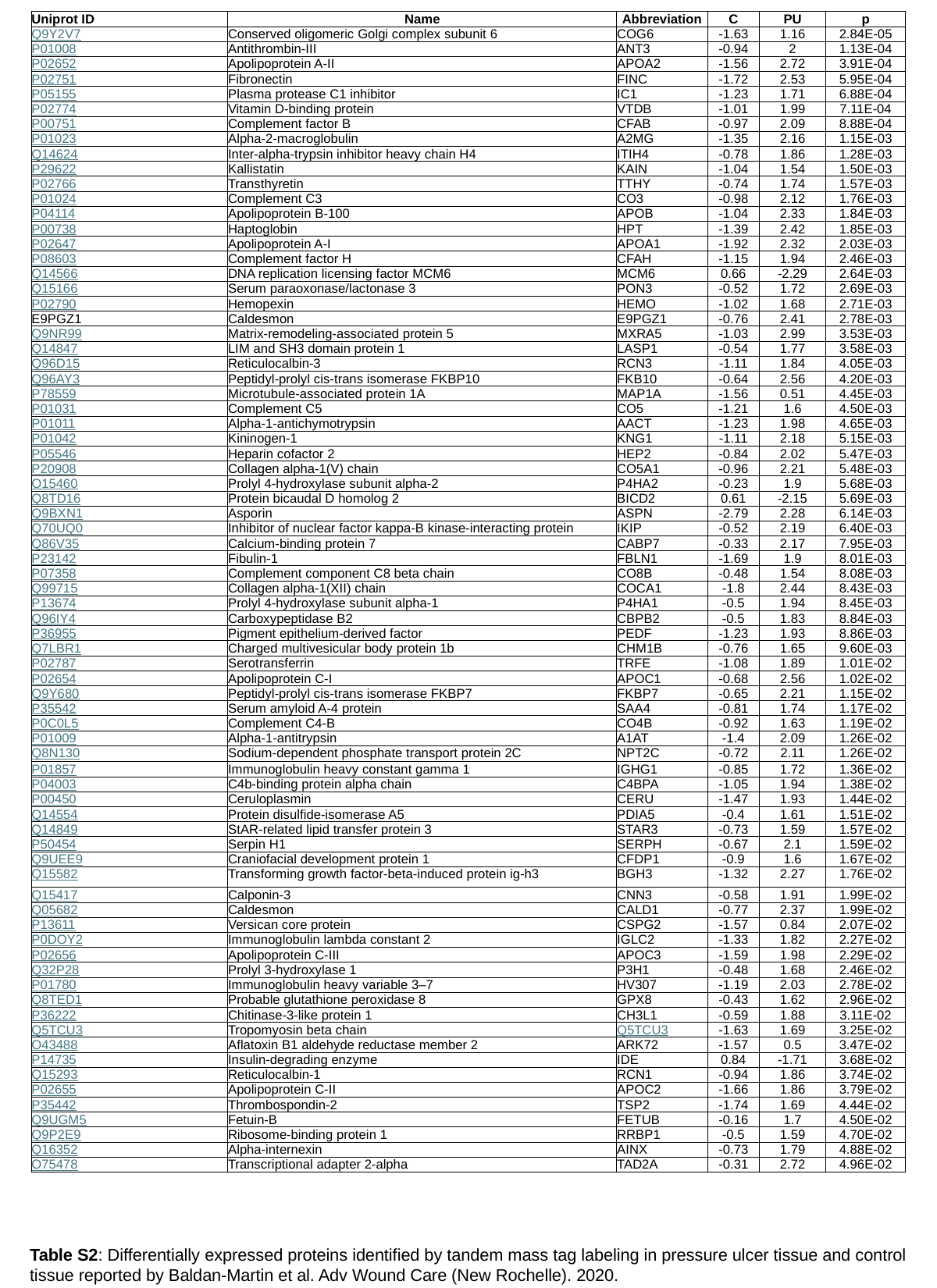

| Uniprot ID | Name | Abbreviation | C | PU | p |
| --- | --- | --- | --- | --- | --- |
| Q9Y2V7 | Conserved oligomeric Golgi complex subunit 6 | COG6 | -1.63 | 1.16 | 2.84E-05 |
| P01008 | Antithrombin-III | ANT3 | -0.94 | 2 | 1.13E-04 |
| P02652 | Apolipoprotein A-II | APOA2 | -1.56 | 2.72 | 3.91E-04 |
| P02751 | Fibronectin | FINC | -1.72 | 2.53 | 5.95E-04 |
| P05155 | Plasma protease C1 inhibitor | IC1 | -1.23 | 1.71 | 6.88E-04 |
| P02774 | Vitamin D-binding protein | VTDB | -1.01 | 1.99 | 7.11E-04 |
| P00751 | Complement factor B | CFAB | -0.97 | 2.09 | 8.88E-04 |
| P01023 | Alpha-2-macroglobulin | A2MG | -1.35 | 2.16 | 1.15E-03 |
| Q14624 | Inter-alpha-trypsin inhibitor heavy chain H4 | ITIH4 | -0.78 | 1.86 | 1.28E-03 |
| P29622 | Kallistatin | KAIN | -1.04 | 1.54 | 1.50E-03 |
| P02766 | Transthyretin | TTHY | -0.74 | 1.74 | 1.57E-03 |
| P01024 | Complement C3 | CO3 | -0.98 | 2.12 | 1.76E-03 |
| P04114 | Apolipoprotein B-100 | APOB | -1.04 | 2.33 | 1.84E-03 |
| P00738 | Haptoglobin | HPT | -1.39 | 2.42 | 1.85E-03 |
| P02647 | Apolipoprotein A-I | APOA1 | -1.92 | 2.32 | 2.03E-03 |
| P08603 | Complement factor H | CFAH | -1.15 | 1.94 | 2.46E-03 |
| Q14566 | DNA replication licensing factor MCM6 | MCM6 | 0.66 | -2.29 | 2.64E-03 |
| Q15166 | Serum paraoxonase/lactonase 3 | PON3 | -0.52 | 1.72 | 2.69E-03 |
| P02790 | Hemopexin | HEMO | -1.02 | 1.68 | 2.71E-03 |
| E9PGZ1 | Caldesmon | E9PGZ1 | -0.76 | 2.41 | 2.78E-03 |
| Q9NR99 | Matrix-remodeling-associated protein 5 | MXRA5 | -1.03 | 2.99 | 3.53E-03 |
| Q14847 | LIM and SH3 domain protein 1 | LASP1 | -0.54 | 1.77 | 3.58E-03 |
| Q96D15 | Reticulocalbin-3 | RCN3 | -1.11 | 1.84 | 4.05E-03 |
| Q96AY3 | Peptidyl-prolyl cis-trans isomerase FKBP10 | FKB10 | -0.64 | 2.56 | 4.20E-03 |
| P78559 | Microtubule-associated protein 1A | MAP1A | -1.56 | 0.51 | 4.45E-03 |
| P01031 | Complement C5 | CO5 | -1.21 | 1.6 | 4.50E-03 |
| P01011 | Alpha-1-antichymotrypsin | AACT | -1.23 | 1.98 | 4.65E-03 |
| P01042 | Kininogen-1 | KNG1 | -1.11 | 2.18 | 5.15E-03 |
| P05546 | Heparin cofactor 2 | HEP2 | -0.84 | 2.02 | 5.47E-03 |
| P20908 | Collagen alpha-1(V) chain | CO5A1 | -0.96 | 2.21 | 5.48E-03 |
| O15460 | Prolyl 4-hydroxylase subunit alpha-2 | P4HA2 | -0.23 | 1.9 | 5.68E-03 |
| Q8TD16 | Protein bicaudal D homolog 2 | BICD2 | 0.61 | -2.15 | 5.69E-03 |
| Q9BXN1 | Asporin | ASPN | -2.79 | 2.28 | 6.14E-03 |
| Q70UQ0 | Inhibitor of nuclear factor kappa-B kinase-interacting protein | IKIP | -0.52 | 2.19 | 6.40E-03 |
| Q86V35 | Calcium-binding protein 7 | CABP7 | -0.33 | 2.17 | 7.95E-03 |
| P23142 | Fibulin-1 | FBLN1 | -1.69 | 1.9 | 8.01E-03 |
| P07358 | Complement component C8 beta chain | CO8B | -0.48 | 1.54 | 8.08E-03 |
| Q99715 | Collagen alpha-1(XII) chain | COCA1 | -1.8 | 2.44 | 8.43E-03 |
| P13674 | Prolyl 4-hydroxylase subunit alpha-1 | P4HA1 | -0.5 | 1.94 | 8.45E-03 |
| Q96IY4 | Carboxypeptidase B2 | CBPB2 | -0.5 | 1.83 | 8.84E-03 |
| P36955 | Pigment epithelium-derived factor | PEDF | -1.23 | 1.93 | 8.86E-03 |
| Q7LBR1 | Charged multivesicular body protein 1b | CHM1B | -0.76 | 1.65 | 9.60E-03 |
| P02787 | Serotransferrin | TRFE | -1.08 | 1.89 | 1.01E-02 |
| P02654 | Apolipoprotein C-I | APOC1 | -0.68 | 2.56 | 1.02E-02 |
| Q9Y680 | Peptidyl-prolyl cis-trans isomerase FKBP7 | FKBP7 | -0.65 | 2.21 | 1.15E-02 |
| P35542 | Serum amyloid A-4 protein | SAA4 | -0.81 | 1.74 | 1.17E-02 |
| P0C0L5 | Complement C4-B | CO4B | -0.92 | 1.63 | 1.19E-02 |
| P01009 | Alpha-1-antitrypsin | A1AT | -1.4 | 2.09 | 1.26E-02 |
| Q8N130 | Sodium-dependent phosphate transport protein 2C | NPT2C | -0.72 | 2.11 | 1.26E-02 |
| P01857 | Immunoglobulin heavy constant gamma 1 | IGHG1 | -0.85 | 1.72 | 1.36E-02 |
| P04003 | C4b-binding protein alpha chain | C4BPA | -1.05 | 1.94 | 1.38E-02 |
| P00450 | Ceruloplasmin | CERU | -1.47 | 1.93 | 1.44E-02 |
| Q14554 | Protein disulfide-isomerase A5 | PDIA5 | -0.4 | 1.61 | 1.51E-02 |
| Q14849 | StAR-related lipid transfer protein 3 | STAR3 | -0.73 | 1.59 | 1.57E-02 |
| P50454 | Serpin H1 | SERPH | -0.67 | 2.1 | 1.59E-02 |
| Q9UEE9 | Craniofacial development protein 1 | CFDP1 | -0.9 | 1.6 | 1.67E-02 |
| Q15582 | Transforming growth factor-beta-induced protein ig-h3 | BGH3 | -1.32 | 2.27 | 1.76E-02 |
| Q15417 | Calponin-3 | CNN3 | -0.58 | 1.91 | 1.99E-02 |
| Q05682 | Caldesmon | CALD1 | -0.77 | 2.37 | 1.99E-02 |
| P13611 | Versican core protein | CSPG2 | -1.57 | 0.84 | 2.07E-02 |
| P0DOY2 | Immunoglobulin lambda constant 2 | IGLC2 | -1.33 | 1.82 | 2.27E-02 |
| P02656 | Apolipoprotein C-III | APOC3 | -1.59 | 1.98 | 2.29E-02 |
| Q32P28 | Prolyl 3-hydroxylase 1 | P3H1 | -0.48 | 1.68 | 2.46E-02 |
| P01780 | Immunoglobulin heavy variable 3–7 | HV307 | -1.19 | 2.03 | 2.78E-02 |
| Q8TED1 | Probable glutathione peroxidase 8 | GPX8 | -0.43 | 1.62 | 2.96E-02 |
| P36222 | Chitinase-3-like protein 1 | CH3L1 | -0.59 | 1.88 | 3.11E-02 |
| Q5TCU3 | Tropomyosin beta chain | Q5TCU3 | -1.63 | 1.69 | 3.25E-02 |
| O43488 | Aflatoxin B1 aldehyde reductase member 2 | ARK72 | -1.57 | 0.5 | 3.47E-02 |
| P14735 | Insulin-degrading enzyme | IDE | 0.84 | -1.71 | 3.68E-02 |
| Q15293 | Reticulocalbin-1 | RCN1 | -0.94 | 1.86 | 3.74E-02 |
| P02655 | Apolipoprotein C-II | APOC2 | -1.66 | 1.86 | 3.79E-02 |
| P35442 | Thrombospondin-2 | TSP2 | -1.74 | 1.69 | 4.44E-02 |
| Q9UGM5 | Fetuin-B | FETUB | -0.16 | 1.7 | 4.50E-02 |
| Q9P2E9 | Ribosome-binding protein 1 | RRBP1 | -0.5 | 1.59 | 4.70E-02 |
| Q16352 | Alpha-internexin | AINX | -0.73 | 1.79 | 4.88E-02 |
| O75478 | Transcriptional adapter 2-alpha | TAD2A | -0.31 | 2.72 | 4.96E-02 |
Table S2: Differentially expressed proteins identified by tandem mass tag labeling in pressure ulcer tissue and control tissue reported by Baldan-Martin et al. Adv Wound Care (New Rochelle). 2020.
